# Acoustic Parametric Signal Generation for Underwater Communication

**DOI:** 10.3390/s18072149

**Published:** 2018-07-04

**Authors:** María Campo-Valera, Miguel Ardid, Dídac D. Tortosa, Ivan Felis, Juan A. Martínez-Mora, Carlos D. Llorens, Pablo Cervantes

**Affiliations:** 1 Institut d’Investigació per a la Gestió Integrada de les Zones Costaneres (IGIC), Universitat Politècnica de València (UPV), 46730 Gandia, València, Spain; didieit@epsg.upv.es (D.D.T.); ivfeen@upv.es (I.F.); jmmora@fis.upv.es (J.A.M.-M.); cdavid@upv.es (C.D.L.); 2 Laboratorio de Hidroacústica (LHA), Centro Tecnológico Naval y del Mar (CTN), Ctra El Estrecho-Lobosillo, Km. 2, 30320 Fuente Álamo, Murcia Spain; pablocervantes@ctnaval.com

**Keywords:** underwater acoustic communication, parametric technique, self-demodulation

## Abstract

This paper presents a study of different types of parametric signals with application to underwater acoustic communications. In all the signals, the carrier frequency is 200 kHz, which corresponds to the resonance frequency of the transducer under study and different modulations are presented and compared. In this sense, we study modulations with parametric sine sweeps (4 to 40 kHz) that represent binary codes (zeros and ones), getting closer to the application in acoustic communications. The different properties of the transmitting signals in terms of bit rate reconstruction, directivity, efficiency, and power needed are discussed as well.

## 1. Introduction

Communications in underwater environments have become a field of research of great interest in recent years. The transmission of information in underwater media can be based on acoustic systems, which present the advantage that the acoustic waves have lower absorption than the electromagnetic ones. However, the underwater acoustic channel has important limitations because of limited bandwidth, extended multipath, severe fading, and refractive properties of the medium. Therefore, it is quite difficult to have clean, direct, and private acoustic communication in underwater environments. To deal with some of these limitations, new methods of communication are proposed based on the non-linear parametric effect. With this technique, directive communication can be achieved by using directive high frequency transducers to produce a low-frequency secondary beam in the medium that can travel over long distances. With this, several advantages are foreseen: to communicate in the desired direction, being more robust against the wanted dissemination of information, or reducing reflections or multi-path effects that could worsen the quality of the communication.

The non-linear parametric effect is observed when a high-intensity acoustic beam with given frequencies is propagated so that secondary frequencies (such as the addition or difference of the primary frequencies) are formed and also propagated. This was first studied by Westervelt [1] and later developed and applied under different conditions [2,3]. The parametric effect has become one of the most popular research areas in underwater acoustics for several decades with multiple applications.

In general, if a modulated emitted wave has a high carrier frequency (primary beam), it self-interferes and is rapidly absorbed in the medium allowing the formation of low frequencies (secondary beam) that propagate at greater distances. As it is well known, it is easier to generate directional beams for high frequencies than for low ones, the latter being usually more omni directional like. However, one of the fundamental characteristics of the parametric effect is that low frequencies, when generated parametrically, have a rather narrow directivity, comparable to that of the primary beam [3].

Theoretical studies have determined that the shape of the secondary beam signal is the second derivative of the square of the emitted signal envelope, its amplitude being proportional to the square of that of the primary beam. The waveform of the secondary beam is determined by the following equation:

(1) $$p\left(x,t\right)=\left(1+\frac{B}{2A}\right)\frac{p^{2}S}{16\pi \rho c^{4}\alpha x}\;\frac{\delta^{2}}{\delta t^{2}}\;{\left[f\left(t-\frac{x}{c}\right)\right]}^{2}\sim \frac{\delta^{2}}{\delta t^{2}}\;f^{2}$$

where *S* is the area of the vibrating surface of the transducer, *f*(*t* − *x/c*)^2^ is the envelope of modulation, *x* is the distance to the source and *t* is time, *B/A* is the nonlinearity parameter of the medium, *ρ* the density, *c* the velocity of sound and *α* the absorption coefficient in the medium. Therefore, the resulting wave *p*(*x*, *t*) is proportional to the second derivative of the envelope of the emitted signal squared [4,5,6].

### Approach

Firstly, this paper proposes a theoretical study in order to optimize the acoustic parameters of an underwater communication system in order to be able to evaluate its performance in terms of sound pressure level and signal-to-noise ratio according to the power and geometry of the acoustic source [7].

Secondly, a study of measured signals is carried out by a plane emitter transducer, determining the relevant characteristics, such as amplitude and directivity of secondary beams with respect to the primary ones. In order to do this, an analysis of the measurements is performed by cross-correlating the emitted signal with the received one and thus obtaining the primary beam [8,9]. To obtain the secondary beam or parametric signal, the received signal is filtered at low frequencies and correlated with the second derivative of the envelope squared of the emitted signal.

Thirdly, the influence of noise in the received signal is studied. The secondary beam is analyzed to determine how the directivity and a proposed Relative Amplitude is affected by noise. All these studies are also interpreted regarding acoustic communication performance in terms of a rate of reconstruction of bits.

## 2. Theoretical Considerations

The level of the secondary beam signal has been obtained following the model by Berktay and Leahy [3]. This model takes into account that the shock wave may limit the control of the secondary beam modulation. It is usually the base for the design of AUV’s communication systems, allowing using simple calculations for circular piston arrays, and other geometries such as square or rectangular shapes [10].

The input parameters of this model are: sound speed c, carrier frequency $f_{p}$, secondary beam frequency $f_{s}$, absorption of the carrier frequency $\alpha_{p}$, absorption of the secondary frequency $\alpha_{s}$, diameter of the transducer d, and the power of the source $W_{o}$. Thus, the design is done according to the following equations (see ref. [7] for more information on the notation and units of the quantities used):

(2) $$S=\left(\pi d^{2}/4\right)\mathrm{Vibrating}\;\mathrm{surface}$$

(3) $$\lambda_{p}=c/f_{p}\;\mathrm{Wavelength}\;\mathrm{of}\;\mathrm{carrier}\;\mathrm{carrier}$$

(4) $$SL_{p}=10\log W_{o}+171+10\log\left(4\pi S/\lambda_{p}^{2}\right)\;\mathrm{Primary}\;\mathrm{beam}\;\mathrm{pressure}\;\mathrm{level}$$

(5) $$SL_{s}=2SL_{p}+20\log\left(fs\right)kHz+20\log\left(\Delta \right)-287\;\mathrm{Secondary}\;\mathrm{beam}\;\mathrm{pressure}\;\mathrm{level}$$

(6) $$\Delta =\int_{0}^{\infty }\frac{e^{-x}}{x+Z}\;dx,\mathrm{where}Z=\alpha_{T}R_{p}\left(\frac{f_{p}}{f_{s}}\right)\;\mathrm{and}\;R_{p}=\left(\frac{S}{\lambda_{p}}\right)\;\mathrm{Effective}\;\mathrm{length}\;\mathrm{of}\;\mathrm{the}\;\mathrm{parametric}\;\mathrm{array}\;\alpha_{T}=2\alpha_{p}-\alpha_{s}\;\mathrm{Total}\;\mathrm{Absorption}$$

(7) $$SL_{c}=20\log L-20\log\left(f_{p}\right)+292\;\mathrm{Critical}\;\mathrm{source}\;\mathrm{level}\;(\mathrm{saturation})$$

(8) $$TL=60+20\log R_{km}+\alpha_{s}R\;\mathrm{Transmission}\;\mathrm{losses}$$

(9) $$NL=60-17\log f_{s}\;\mathrm{Noise}\;\mathrm{level}$$

(10) $$DI=10(4\pi S/\lambda_{s}^{2})\;\mathrm{Directivity}\;\mathrm{and}\lambda_{s}\;\mathrm{wavelenght}\;\mathrm{of}\;\mathrm{secondary}\;\mathrm{beam}$$

(11) $$SNR=SL_{s}-TL-NL+DI-10\log B\;\mathrm{Signal-to-noise-ratio}$$

### Transmitter Acoustic Response

As an example, these equations are applied to the emitter Airmar P19 transducer used in this paper, whose active element is a cylindrical piezoelectric ceramic with a diameter of 0.033 m working at the thickness resonant frequency of 200 kHz. The results for a secondary beam of 40 kHz with 1 kHz signal bandwidth at a distance of 10 km are presented in Table 1.

It can be observed that, for our transducer, the value for *SNR* is very high. This is because of the assumptions on the noise level (it may be considerably higher in some real situations) or on the transmission loss [10]. Even so, with this example we can show the potential of the parametric array concept, serving as the basis for the design of the application in underwater acoustic communication.

## 3. Experimental Set-Up

**Figure 1:**
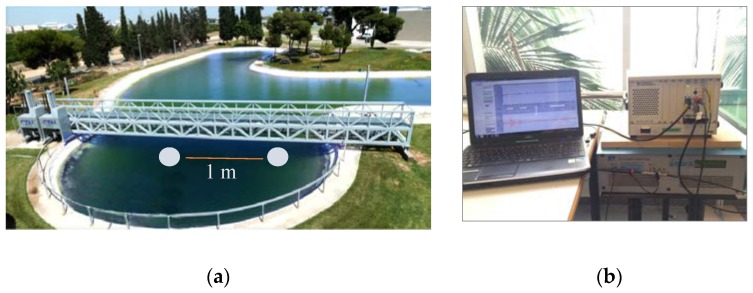
Pictures of the experimental setup. (**a**) The lake where the position of transducers is indicated with white circles. (**b**) Equipment used for the calibrations and measurements.

The measurements were made in the Centro Tecnológico Naval y del Mar (CTN) in Murcia, Spain, in a lake of tapered shape with a 10 m depth and a diameter of 20 m. Figure 1a,b are pictures of the experimental setup. The distance between the emitter and the receiver was 1 m. An ITC 1032 transducer was used as a receiver with a receiving sensitivity (RVR) of −194 dB re 1 V/μPa, without much variation at the resonance frequency region at 33 kHz and below, and thus was quite sensitive to the low frequencies willing to be detected. The Airmar P19 plane transducer was chosen as acoustic transmitter. Figure 1b shows the equipment used in the measurements. The transmitter transducer was driven through the National Instruments 5412 PXI signal generator with a 50 dB gain using the E&I 2100L RF amplifier. Signal reception data taking was done through the PXI National Instruments 5102 card. A sampling frequency $f_{s}=20\;\mathrm{MHz}$ was used for data acquisition. The position of the transducers was set using a positioning system controlled by brushless motors with axis *X* = servo motor BMH1003P32F2A with gearbox, axis *Z* = servo motor BMH1003P32F2A axis PAN (flat pan turn) = servo motor BSH0553P01A2A axis tilt (vertical flat turn) = servo motor BSH0553P01A2A.

### Characteristics of the Transmitter

**Figure 2:**
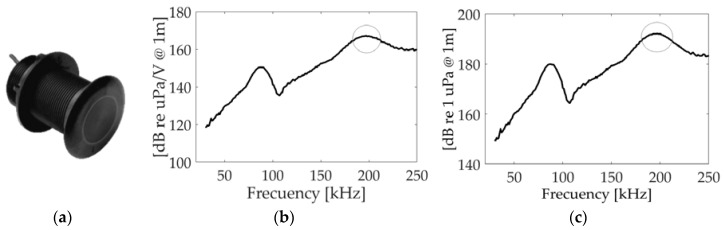
Airmar P19. (**a**) Picture; (**b**) Transmitting Voltage Response (TVR); (**c**) SPL, highlighting the interesting 200 kHz region for this application.

The knowledge of the transmitter behavior is essential for the proposed application. Figure 2b, shows the Transmitting Voltage Response (TVR), which is the ratio of the pressure signal emitted to the applied voltage, for Airmar P19. It has been measured at the lab using tone bursts at different frequencies. The carrier frequency $f_{o}$ of the modulation signal corresponds to the resonance frequency at 200 kHz and the TVR at this frequency is 167 dB re µPa/V@1 m. In Figure 2c, the values of sound pressure level reached in our experiment are presented, with a value for the frequency of 200 kHz of 195 dB re µPa@1 m. The directivity of the transducer is another important parameter for the proposed study. The transducer presents a beamwidth (@−3 dB) at 200 kHz of 11°.

## 4. Results

### 4.1. First Studies

**Figure 3:**
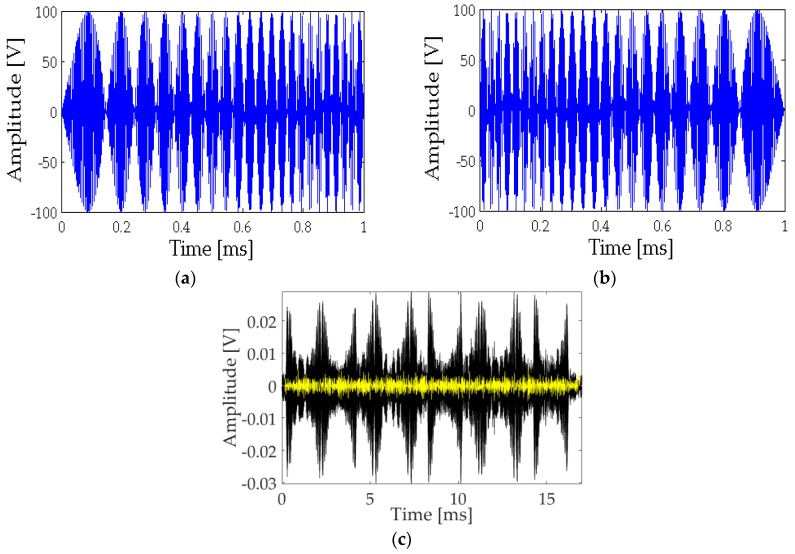
Signal used in the analysis. (**a**) Bit 1: Upwards sine sweep signal; (**b**) Bit 0: Downwards sine sweep signal; (**c**) 16-bit received signal (black) and filtered at low frequencies (multiplied by a factor of 20, yellow).

The study for the parametric generation of a sine sweep signal with the emitting transducer was carried out by emitting frequency-modulated signals with carrier frequency of 200 kHz. Two modulated sine sweep signals were used as basic codification of the information: bit 1, upwards from 4 to 40 kHz, and bit 0, downwards from 40 to 4 kHz, corresponding to Figure 3a,b, respectively.

Figure 3c shows the received signal being a mixture of the primary beam at 200 kHz and the secondary beam at low frequency produced by the parametric effect. In order to distinguish the secondary beam, a bandpass filter (2 to 42 kHz) was applied. The secondary beam (yellow) is multiplied by a factor of 20 to be visible along with the original received signal.

A parametric sine sweep signal with a frequency bandwidth of 4 to 40 kHz with a duration of 1 ms and a carrier frequency $f_{o}=200\;\mathrm{kHz}$ is then used for the communication study. The idea is to generate a 16-bit string = 1010010110010110, code of ones and zeros with this signal; where bit 1 corresponds to the sine sweep signal from 4 to 40 kHz and bit 0 corresponds to a sine sweep signal from 40 to 4 kHz. Using cross correlation techniques, it is possible to recognize the parametric signal since the correlation produces a clear narrow peak on the signal arrival time, which allows it to be distinguished from near echoes, and increases the signal to noise ratio [8,11].

The signals emitted for bit 1 and bit 0, as well as the received signal for the string are shown in Figure 3.

**Figure 4:**
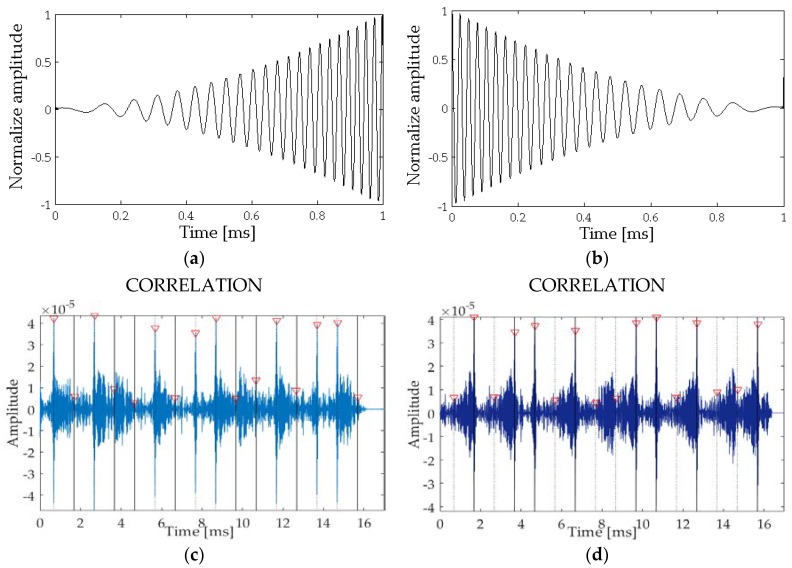
Signal analysis using cross-correlation. (**a**) second time derivative of envelope for bit 1; (**b**) second time derivative of envelope for bit 0; (**c**) Cross correlation signal with bit 1; (**d**) Cross correlation signal with bit 0.

The expected secondary beams, that is, the second derivative of the envelope to the square of the signals emitted are presented in Figure 4a,b for bits 1 and 0, respectively.

Figure 4c,d show the results of the cross-correlation between the received signal filtered at low frequency containing the 16-bits string (Figure 3c, yellow) with the expected secondary beams for bits 1 and 0 (Figure 4a,b). Thus, one can observe that correlating with bit 1 the cross-correlation amplitudes for the positions of bit 1 of the string are much greater than the corresponding amplitudes for bit 0. In the same way, the positions of bit 0 are clearly enhanced in the correlation amplitudes when correlating with bit 0.

Obtained the amplitude voltage by cross-correlation for the detection of bit 1 and 0, presented in Figure 4c,d, and specified in Table 2, a parameter, called Relative Amplitude RA, is introduced for bit detection analysis. It is defined as:

(12) $$RA=\frac{V-F}{MAX\left(V;F\right)}$$

where V is the amplitude for the true bit, that is; the one that should be in correlation with the bit emitted (1 or 0) and F is a false bit since it is the bit not being emitted. In this sense, if the bit is detected correctly, the value V will be greater than F and therefore the RA values will be between 0 and +1. On the other hand, if RA is between 0 and –1, it means a wrong detection.

In Table 2 the results for a direct communication for transducers at 1 m distance, facing each other, so at 0 degrees, are presented. A detection time of about 0.63 ms is obtained. The Relatives Amplitudes RA obtained after the cross-correlation and the assigned bit are also presented, showing that the information could easily be extracted. In this sense the RA is correct for each bit position, oscillating between 0.78 and 0.86, so close to +1.

**Figure 5:**
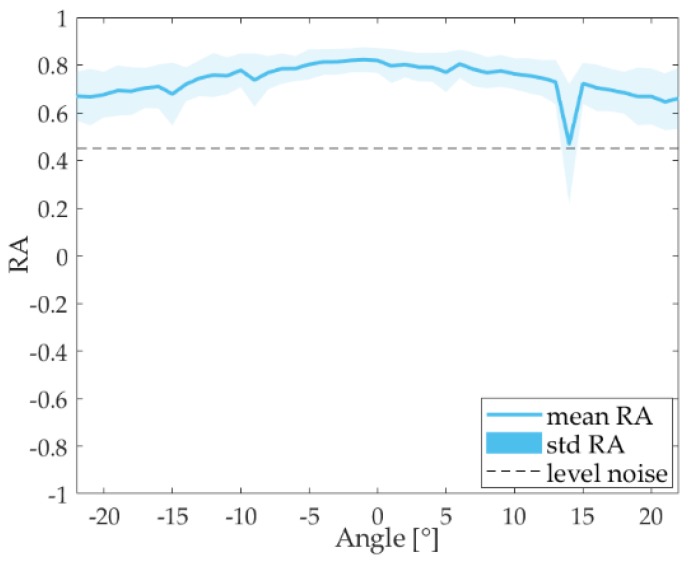
Relative amplitude for string bits.

In Figure 5, the *RA* parameter averaged for all bit positions is presented as a function of the directivity angle of the emitter. We can observe that for this case all *RA* values are above the noise level region, which is between ±0.45, so the detection is produced for all angles (± 25°).

### 4.2. Parametric Studies

To confirm the non-linear parametric effect and see the applicability of this technique for communication purposes, three different studies have been performed:

1. Attenuation—as a function of distance.
2. Voltage variation—as a function of the primary beam intensity.
3. Directivity—as a function of the angle of emission.

The first study analyzes the secondary beam generation in the medium by changing the distance between the emitter and the receiver. The second one aims to compare the amplitudes of the primary and secondary beams by emitting the signal at low intensity (that is, low feeding voltage) and increasing it to demonstrate the non-linear effect. The third study compares the directivity pattern of both beams.

**Figure 6:**
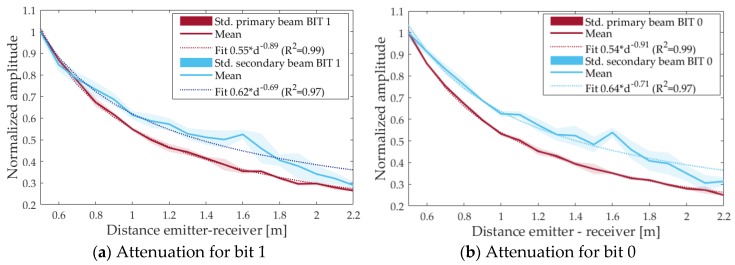
Normalized amplitude of the received signal for primary (red) and secondary (blue) beam as a function of distance between emitter and receiver. Std means the standard deviation. (**a**) for bit 1; (**b**) for bit 0.

Figure 6 shows the results varying the distance, d, between the emitter and the receiver from 0.50 to 2.2 m, in steps of 0.10 m. The measurements of both, primary and secondary beams, are adjusted to a function $a\cdot d^{-b}$. Neglecting the absorption, for a spherical propagation beam, a value of b = 1 is expected. The value for the primary beam is 0.89, so close to 1, however the value for the secondary beam is much smaller, 0.69, and this can be understood as a hint of the parametric generation of the beam in the medium, and therefore there is less attenuation.

**Figure 7:**
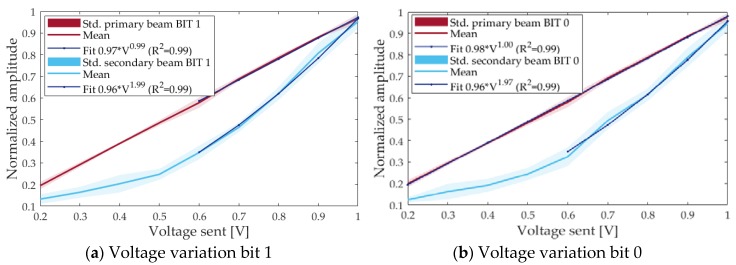
Normalized amplitude of the received signal for primary beam (red) and secondary beam (blue) as a function of voltage sent of emitted signal (i.e., feeding voltage before the amplifier). Std. means the standard deviation. (**a**) for bits 1; (**b**) for bits 0.

The dependence with respect to the intensity of the primary beam was done by setting the amplitude in the waveform signal generator from 200 mV to 1 V in steps of 100 mV, and studying the received amplitude. As shown in Figure 7, a fit with the function $a\cdot x^{b}$ was made with data from 600 mV to 1 V. An exponent of 0.99 (linear behavior) is obtained for the primary beam whereas an exponent of 1.98 (square behavior) is obtained for the secondary beam, so agreeing perfectly to the theory of parametric emission.

**Figure 8:**
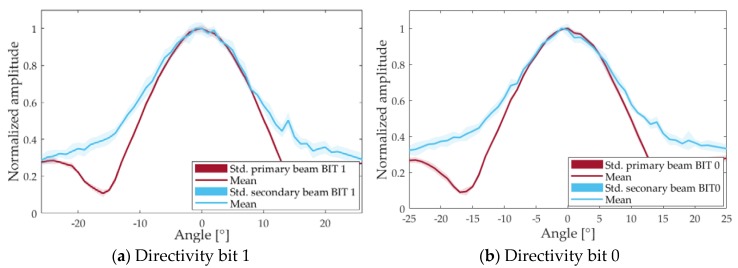
Directivity pattern of the primary and secondary beams.

The evidence of the parametric effect of the secondary beam is also clearly shown in the directivity study, the results of which are presented in Figure 8. An open angle of ±12° is obtained for the secondary beam, whereas for the primary beam, it is ±10°. So, both beams present a quite similar directivity pattern despite the big differences in the frequency content.

Summarizing, all these effects agree that the signal has been generated parametrically and thus, this technique could be used for underwater acoustic communications in circumstances where highly directive beams are preferable.

### 4.3. Influence of Noise

In this section, we study the influence of the noise on the communication using the parametric technique. To this end, a study is carried out that consists of adding to the received signals a white noise with different amplitude values ranging from 1 to 10 mV. Then, the correlation process described in Section 4.1 is performed and the parameters *RA* and *RB*, Reconstructed bits, are analyzed.

**Figure 9:**
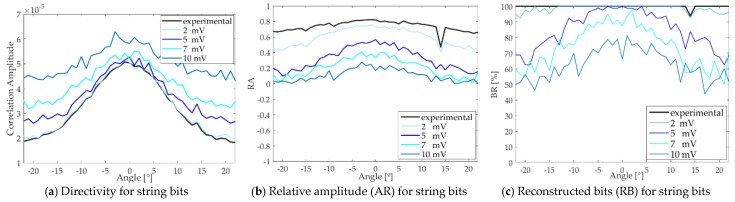
Influence of the noise in the secondary beam for the string of bits (experimental) and the ones adding some white noise of different amplitude: 2, 5, 7 and 10 mV. (**a**) Directivity; (**b**) Relative amplitude; (**c**) Reconstructed bits.

We can see in Figure 9 that by increasing the amplitude of the white noise the directivity is degraded (a), the *RA* parameter decreases (b) and, so the bit detection rate also does (c). Therefore, the signal is sensitive to changes in amplitude of noise and *RB* is quite dependent of the angle. For example, with 5 mV white noise *RB* is larger than 90% in the region ±10°, whereas it degrades quite fast outside this region. This could be applied for situation in which directive communication is required. The noise could either be environmental or easily produced artificially with a non-directive transducer working at low frequencies.

### 4.4. Amplitude Ratio Between the Secondary and Primary Beams

In this section we compare the amplitude ratio between the secondary beam and the primary beam $A_{s}/A_{p}$ obtained experimentally with a theoretical model referenced in [2].

A 0.28% direct ratio has been measured for the pressure amplitude of both beams, 12,940 Pa for the primary beam and 36.8 Pa for the secondary one.

With respect to the theoretical predictions, using the first approach, the equation of the complex parametric gain *G* (dB) is used, which is defined as a value whose magnitude is the ratio between the primary and secondary beam pressures [2], where.

(13) $$g\equiv rP\left(r,0\right)/R_{o}P_{o}$$

and in decibels:

(14) G=20log|g|

where r is the observer’s radial coordinate, P(r,0) secondary beam pressure at a point *r* centered on the emitter (Pa), $R_{o}=A_{o}f_{o}/c$ is the Rayleigh length (m), $P_{o}$ the peak face pressure amplitude of the primary component (Pa) at the Rayleigh distance.

According to the specifications of the emitters and the calibrations done, for our case we have g≈0.0028 and G≡−51 dB, thus obtaining an amplitude ratio between the secondary beam and the primary beam of 0.28%, so fully agreeing with the measurements. This gain G corresponds to a value of $L_{o}^{*}=245$ dB rms source level of the primary component and $\bar{\alpha }R_{o}\approx 10^{-3}$ dB (0.0012 dB).

(15) $$Lo=20\log(P_{o}R_{o}/\sqrt{2}),\;\mathrm{and}\;L_{o}^{*}=L_{o}+20\log f_{o}(\mathrm{db}//1\;\mu \mathrm{Pa}\;m\;\mathrm{kHz})$$

According to the parametric-gain curves, Figure 2 of Ref. [2], we effectively find values close to $10^{-3}$ for $\bar{\alpha }R_{o}$ and $L_{o}^{*}\;\sim \;240$ in the shown curves.

Moreover, by applying Equation (1), the measured sweep is studied with a set of parametric sine waves at the limit frequencies and a centered one; that is, 4 kHz, 20 kHz and 40 kHz frequencies are chosen. The ratios between the secondary beam and the primary beam obtained are 0.141‰, 0.35% and 1.41%, respectively, with a primary beam pressure of 12,940 Pa. As expected, the closest ratio to the experimental one is the one for 20 kHz sine wave, which is about the average frequency of the parametric sweep emitted.

Finally, the measurement is also contrasted according to the operating regime for parametric sources. Following ref. [2], our case is in the regime of absorption limiting in the far spherical zone:

(16) $$X\ll 1,\;2\alpha R_{o}f_{o}/f\ll 1$$

where α(Np/m) is the absorption, $R_{o}\left(m\right)$ Rayleigh length, $f_{o}$ (Hz) carrier frequency and f difference frequency 4, 20 and 40 kHz. Applying the Equation (16) for $2\alpha R_{o}f_{o}/f$ we obtain 0.014, 0.0028 and 0.0014, respectively, and for X 0.006, so all values are much smaller than one.

Subsequently, the parameter g is calculated through the following equation:

(17) $$\left|g\right|=\frac{X}{2}\;\frac{f}{f_{o}}\;E_{1}\left(\frac{2\alpha R_{o}f_{o}}{f}\right)$$

where $E_{1}$ is the exponential integral function. We obtain 0.0002 for 4 kHz, 0.0015 for 20 kHz and 0.0035 for 40 kHz and applying Equation (14) we have −73.36 dB −56.27 dB and −49.18 dB, respectively, validating that for the emitted frequency of 20 kHz the value of *G* = −56.27 dB is similar to the first analysis studying the pressure received from the primary and secondary beams with *G* = −51 dB.

## 5. Conclusions

The generation and analysis of parametric signals for a plane emitter transducer has been discussed in order to apply it to underwater acoustic communications. The formulations presented to optimize the design of an array according to the model of Berktay and Leahy lay the foundations for developing the design of the experiment. The dependence of the parametric signal with respect to the primary beam intensity, attenuation, and directivity, as well as the corresponding impact in communications in terms of bit reconstruction has been studied and evaluated. With respect to this, we can conclude that the parametric generation allows a better use of the communication channel, which allows transmitting in a more defined region, thus allowing a more private communication, or not adding acoustic contamination to protected areas. Moreover, for some cases, this method helps to improving the resistance against possible background noise and interference.

On the other hand, the rapid absorption of high frequencies in the medium allows the low frequencies (secondary beam) to propagate at greater distances with a rather narrow directivity angle of the order of 10° for a frequency bandwidth between the 4 kHz and 40 kHz, as presented in this study. This is a very remarkable difference with respect to the use of conventional transducers, which usually present a directivity angle of ~60°.

As future work, we are considering to extent the of study of the modulation signals by varying frequency ranges and time duration, so as to increase the communication speed and minimize the bit-error rates. Moreover, other kinds of modulations used in acoustic communication could also be explored for the parametric case. We are also considering the study of the application of the technique in shallow waters since the parametric beam is quite narrow to verify that the multipath effect is minimized with respect to the case of linear communications, much less directive. And finally, the application in situations where some specific region should not be exposed to the acoustic waves and messages due to either privacy or environmental reasons.

## Figures and Tables

**Table 1:** Results for the beams at a distance of 10 km.

*f_s_* [kHz]	Power [W]	*TL*@10 km [dB]	*NL* [dB]	*DI* [dB]	*SLp* [dB]	*SLc* [dB]	*SLs* [dB]	*SNR*@1 kHz [dB]
40	182	92	33	9	216	225	180	34

**Table 2:** Parameters of the detection and interpretation of the 16-bit signal received for transducers facing each other at 1 m distance.

Bit Position	Bits	Detection Time [ms]	Amplitude BIT 1 [10^−4^]	Amplitude BIT 0 [10^−4^]	*RA*
1	1	0.634	0.4069	0.066	0.8378
2	0	1.692	0.0661	0.3846	0.8281
3	1	2.694	0.4198	0.0642	0.8471
4	0	3.693	0.0776	0.3736	0.7923
5	0	4.692	0.0629	0.393	0.8399
6	1	5.653	0.3895	0.0516	0.8675
7	0	6.693	0.0747	0.3739	0.8002
8	1	7.631	0.3847	0.0593	0.8459
9	1	8.642	0.4056	0.0903	0.7774
10	0	9.693	0.0787	0.3805	0.7932
11	0	10.69	0.0874	0.4126	0.7882
12	1	11.69	0.4084	0.0704	0.8276
13	0	12.69	0.08	0.4066	0.8032
14	1	13.69	0.4014	0.073	0.8181
15	1	14.69	0.4251	0.0822	0.8066
16	0	15.69	0.0538	0.3898	0.8620
